# Supplementary material for: Survey of Clinical Practice Patterns of Korean Medicine Doctors for Anorexia in Children: A Preliminary Study for Clinical Practice Guidelines
Source: Children (Basel). 2022 Sep 17;9(9):1409. doi: 10.3390/children9091409 (PMC9497536; doi:10.3390/children9091409)
Supplement: Supplementary file 1 [file children-09-01409-s001.zip › children-1909169-supplementary.pdf]

Table S1. Survey of Clinical Practice Patterns of Korean Medicine Doctors for Anorexia in Children

Ⅰ. Current status of treatment for anorexia in children

1. Based on the last month, what is the monthly average number of first-time patients with anorexia in children?

① ≤ 5

② 6–10

③ 11–20

④ 21–30

⑤ 31–40

⑥ ≥ 41

2. Based on the last year, what is the average treatment period for children with anorexia?

① < 1 month

② 1 month or more and less than 3 month

③ 3 months or more and less than 6 months

④ 6 months or more and less than 1 year

⑤ 1 year or more and less than 3 year

⑥ ≥ 3 years

3. Based on the previous year, what is the average treatment cost (copay) for children with anorexia? If decoction is included, it is calculated by dividing the total cost of taking it by the number of days of treatment.

① < 5,000

② 5,000 or more and less than 10,000

③ 10,000 or more and less than 20,000

④ 20,000 or more and less than 50,000

⑤ 50,000 or more and less than 100,000

⑥ ≥ 100,000

4. What is the main age range for children with anorexia? (Select all that apply)

① Infants (1 month–1 year)

② Preschool children (1–7 years)

③ Children in early elementary school (7–9 years)

④ Children in the upper grades of elementary school (10–12 years)

⑤ Middle school students (13–15 years)

⑥ High-school students and above (≥ 16 years)

Ⅱ. Diagnosis

**Diagnosis of Infantile Anorexia (IA),**
The child (a) refuses to eat adequate amounts of food for at least 1 month and shows growth deficiency, (b) does not communicate hunger and lacks interest in food, and (c) the child's food refusal does not follow a traumatic event and is not due to an underlying medical illness.
Reference: Diagnostic Classification of Mental Health and Developmental Disorders of Infancy and Early Childhood Revised; DC: 0–3R

1. In the case of children over three years of age, there were no clear diagnostic criteria. Do you agree to extend the above criteria to the full age range of children (age 0–19 years)?

① Yes. (I agree to extend to 0–19 years)

② No. (It is not appropriate to extend to 0–19 years).

③ I don't know.

☞ **When selecting '②' in item 1**

1-2. If you disagree with extending the criteria for anorexia in children to all age groups, please provide your comment (optional).

2. What is the diagnostic method used for children with anorexia? (Select all that apply)

① Diagnosis based on the clinical features

② pattern identification–Qi, Blood, Fluid, Humor, and Organ system diagnosis (氣血津液臟腑辨證) based on KM textbooks

③ pattern identification–Six-Meridian pattern identification (六經辨證) based on “Cold Damage Medicine (傷寒醫學)”

④ pattern identification–Sasang constitutional (四象醫學) diagnosis

⑤ pattern identification–Hyungsang Constitutional medicine (形象醫學)

⑥ pattern identification–Eight Constitutional medicine (八體質)

⑦ Korean Children's Eating Behavior Questionnaire (K-CEBQ)

⑧ KM diagnostic device

⑨ Blood and urine test

⑩ Other (specify :   )

☞ **When selecting ‘③’ in item 2.**

2-1. What are the names of pattern identification used for children with anorexia? (Select all that apply)

① Milk and food damage (乳食傷)

② Spleen failing in transportation (脾失健運)

③ Stomach yin deficiency (胃陰不足)

④ Spleen-stomach qi deficiency (脾胃氣虛)

⑤ Liver depression (肝鬱)

⑥ Others (specify :   )

3. What diagnostic equipment is being used for children with anorexia? (Select all that apply)

① None

② Height and Weight Measurements

③ Bioelectrical impedance analysis (InBody).

④ KM diagnostic device: heart rate variability

⑤ KM diagnostic device–Yangdorak (良導絡) Diagnosis

⑥ KM diagnostic device–Electro Pulse Graph (脈傳導)

⑦ KM diagnostic device: Tongue diagnosis

⑧ KM diagnostic device–Iris Diagnosis

⑨ KM diagnostic device–Constitutional Diagnosis

Digital infrared thermal imaging (DITI)

⑪ Blood and urine test

⑫ Others (specify :   )

Ⅲ. Treatment

1. What is the main treatment for children with anorexia? (Select all that apply)

① Herbal medicine

② Acupuncture

③ Electroacupuncture

④ Moxibustion

⑤ Cupping therapy

⑥ Pharmacopuncture

⑦ Manipulation / Exercise therapy

⑧ Dietetic therapy

⑨ Others (specify :   )

☞ **When selecting '①' in Item 1**

1-1. What types of herbal medicine formulations are mainly used for children with anorexia? (Select all that apply)

①Compound herbal decoction

② Distillation of the compound herbal decoctions

③ Powder preparation

④ Pill preparation

⑤ Mixture of soluble granules covered by insurance.

⑥ Soft extract covered by insurance.

⑦ Mixture of soluble granules not covered by insurance.

⑧ Soft extract not covered by insurance

⑨ Others (specify :   )

☞ **When selecting '①' in Item 1**

1-2. What is the name of the herbal medicine prescription mainly used for patients with anorexia in children? (select all that apply)

① Hyangsayukgunja-tang (香砂六君子湯)

② Jeonssiigong-san (錢氏異功散)

③ Samryeongbaekchul-san (蔘苓白朮丸)

④ Samchulgeonbi-tang (蔘朮健脾湯)

⑤ Hyangsayangwi-tang (香砂養胃湯)

⑥ Bojungikgi-tang (補中益氣湯)

⑦ Taehwa-hwan (太和丸)

⑧ Sogunjung-tang (小建中湯)

⑨ Yangwijeungaek-tang (養胃增液湯)

⑩ Insamyangwi-tang (人蔘養胃湯)

⑪ Bihwa-eum (比和飮)

⑫ Haeulgeonbi-tang (解鬱健脾湯)

⑬ Gokmaekjichul-hwan (曲麥枳朮丸)

⑭ Gyebiwon (啓脾元)

⑮ Sosik-hwan (消食丸)

⑯ Hoojang-hwan (厚腸丸)

⑰ Hyanggyulbyeong (香橘餠)

⑱ Bohwa-hwan (保和丸)

⑲ Chilseong-hwan (七聖丸)

⑳ Others (specify :)

☞ When selecting '①' in Item 1

1-3. When treating children with anorexia with herbal medicines, if herbal medicines are frequently used, what are they? (Select all that apply)

① Atractylodis Rhizoma Alba (白朮)

② Crataegii Fructus (山査)

③ Poria (茯苓)

④ Citri Pericarpium (陳皮)

⑤ Glycyrrhizae Radix (甘草)

⑥ Massa Medicata Fermentata (神麯)

⑦ Hordei Fructus Germiniatus (麥芽)

⑧ Atractylodis Rhizoma (蒼朮)

⑨ Galli Stomachichum Corium (鷄內金)

⑩ Others (specify :   )

☞ When selecting '①' in Item 1

1-4. What is the recommended average duration of herbal medicine treatment for children with anorexia?

① < 1 month

② 1 month or more and less than 3 months

③ 3 months or more and less than 6 months

④ 6 months or more and less than 1 year

⑤ 1 year or more and less than 3 year

⑥ ≥ 3 years

☞ When selecting '②' in Item 1

1-5. What is the most commonly used acupuncture method for children with anorexia? (Select all that apply)

① Meridian points acupuncture (經穴鍼術)

② Sa-am acupuncture therapy (舍巖鍼法)

③ Five element acupuncture (五行鍼法)

④ Constitutional acupuncture (體質鍼法)

⑤ Auricular acupuncture

⑥ Intradermal acupuncture (皮內鍼)

⑦ dermal acupuncture (皮膚鍼)

⑧ Others (specify :)

☞ **When selecting '②' in Item 1**

1-6. Which acupuncture points are mainly used for acupuncture in children with anorexia? (Select all that apply)

① EX-UE10 (四縫)

② ST36 (足三里)

③ CV12 (中脘)

④ CV4 (關元)

⑤ ST25 (天樞)

⑥ PC6 (內關)

⑦ ST24 (滑肉門)

⑧ BL20 (脾兪)

⑨ BL21 (胃兪)

⑩ BL18 (肝兪)

⑪ ST23 (太乙)

⑫ GV4 (命門)

⑬ BL23 (腎兪)

⑭ GV14 (大椎)

⑮ SP6 (三陰交)

⑯ Others (specify :)

☞ **When selecting '④' in Item 1**

1-7. Which acupuncture point is mainly used for moxibustion treatment for children with anorexia?

☞ **When selecting '⑧' in Item 1**

1-8. What is the most commonly used type of dietetic therapy for children with anorexia? (Select all that apply)

① Dietetic therapy based on KM theory (e.g., diet based on constitution)

② Dietetic therapy based on nutritional science (e.g., traffic light diet)

③ Other (Please specify:)

Ⅳ. Perception of Korean medicine treatments

1. Based on your treatment experience, what do you think is the overall effect of Korean medicine treatment for children with anorexia?

Not at all effective - Mostly not effective - Moderate–Mostly effective - Very effective
(5-point Likert scale)

2. What do you think are the advantages of Korean medicine treatment for children with anorexia? (Select all that apply)

① Effective

② Fewer side effects

③ Economical

④ More helpful than other treatment methods.

Fundamental treatment to improve digestive function

⑥ Other (Please specify:)

3. What do you think needs to be supplemented in Korean medicine treatment for children with anorexia? (Select all that apply)

① Treatment cost

② Therapeutic effect

③ Duration of treatment

④ Convenience of treatment (herbal medicine and acupuncture)

⑤ Treatment information (promotion and awareness)

Ⅴ. Safety and effectiveness of Korean medicine treatments

1. How often is the efficacy evaluation performed in the treatment of anorexia in children?

① 1 month

② 3 month

③ 6 month

④ 1 year

⑤ Other (Please specify:)

2. What are the efficacy evaluation indicators in the treatment of anorexia in children? (Select all that apply)

Numerical Rating Scale (NRS)

② Amount of food

③ Weight

④ Height

⑤ Body mass index (BMI)

⑥ Improvement in the general condition of the children

⑦ Other (Please specify:)

3. How often is the safety evaluation performed in the treatment of anorexia in children?

① 1 month

② 3 months

③ 6 months

④ 1 year

⑤ Other (Please specify:)

4. What are the safety evaluation indicators for the treatment of anorexia in children? (Select all that apply)

① Evaluation of Adverse Reactions

② Change in vital signs

③ Changes in the child's general condition

④ Blood test

⑤ Urine test

⑥ Other (Please specify:)

5. Are there any cases of adverse reactions during treatment with herbal medicine for anorexia in children?

① No herbal medicine treatment for children with anorexia.

② No adverse reactions were noted.

③ Adverse reactions were also observed.

☞ **When selecting '③' in item 5.**

5-1. What were the adverse reactions that occurred during treatment with herbal medicine for anorexia in children?

6. Have there been any adverse reactions to acupuncture treatment for anorexia in children?

① No acupuncture treatment for children with anorexia.

② No adverse reactions were noted.

③ Adverse reactions were also observed.

☞ **When selecting ③ in item 6**

6-1. What were the adverse reactions that occurred during acupuncture treatment for anorexia in children?

7. Have there been any adverse reactions to Electroacupuncture treatment for anorexia in children?

①No electroacupuncture treatment for children with anorexia.

②No adverse reactions were noted.

③Adverse reactions were also observed.

7. Have there been any adverse reactions to moxibustion treatment for anorexia in children?

① No moxibustion treatment for children with anorexia.

② No adverse reactions were noted.

③ Adverse reactions were also observed.

☞ **When selecting '③' in item 7.**

7-1. What were the adverse reactions that occurred during moxibustion treatment for anorexia in children?

VI. The need for information to be included in the clinical practice guidelines for Korean medicine treatment

1. What information would you like to know more about to treat anorexia in children? (Up to 3 multiple responses allowed)

① Diagnostic criteria

② Evaluation method

③ Western medicine treatments

④ Treatment with Korean medicine

⑤ Differential diagnosis

⑥ Management

[2-6] When developing the standard clinical practice guidelines for anorexia in children for Korean medicine doctors, please select all the essential information (select all that apply).

2. Diagnostic criteria for anorexia in children
Diagnostic criteria for Western medicine
Diagnostic criteria for Korean medicine (pattern identification)
③ Other (Please specify:)

3. Evaluation method for anorexia in children
① Questionnaire used to assess symptom severity

② Questionnaire for pattern identification

③ Food/Symptom Diary

④ Examination using diagnostic equipment.

⑤ Other (Please specify:)

4. Western medicine treatment for anorexia in children
① Efficacy/mechanism of individual Western medicines.

② Common adverse events and remedies

③ Other (Please specify:)

5. Korean medicine Treatment methods for anorexia in children
① Herbal medicine

② Acupuncture

③ Electroacupuncture

④ Moxibustion

⑤ Pharmacopuncture

⑥ Manipulation/Exercise Therapy

⑦ Dietetic therapy

⑧ Other (Please specify:)

6. Differential diagnosis for anorexia in children

① Information on the types of similar diseases

② Specific information on similar diseases

③ Other (Please specify:)

Ⅶ. Demographic information

1. Are you:

① Female

② Male

2. Your age:

① 20–29

② 30–39

③ 40–49

④ 50–59

⑤ ≥ 60

3. Years of clinical experience:

① ≤4

② 5–9

③ 10–19

④ 20–29

⑤ ≥30

4. Where is your affiliated institution located?

① Seoul Metropolitan City.

② Metropolitan city (□ Busan, □ Daegu, □ Gwangju, □ Incheon, □ Daejeon)

③ Province (□ Gyeonggi-do □ Gangwon-do □ Chungcheongbuk-do)

□ Chungcheongnam-do □ Jeollabuk-do □ Jeollanam-do Province

□ Gyeongsangbuk-do □ Gyeongsangnam-do Province □ Jeju-do

□ Sejong Metropolitan Autonomous City)

5. Specialist training:

① No

② Yes

5-1) Specialty (if applicable)

① Internal Korean Medicine

② Korean Medicine, Obstetrics and Gynecology

③ Pediatrics in Korean Medicine

④ Korean Medicine Neuropsychiatry

⑤ Korean Medicine Ophthalmology, Otolaryngology, and Dermatology

⑥ Korean Medicine Rehabilitation

⑦ Korean acupuncture and moxibustion medicine

⑧ Sasang Constitutional Medicine

6. What is your affiliated institution?

① Korean medicine clinics (primary healthcare institutions)

② Specialized Korean medicine clinics (Korean medicine clinics that focus on specialized treatment for specific diseases)

③ Korean Medicine Hospital (not a university hospital)

④ university teaching Korean medicine hospitals

⑤ convalescent hospital

⑥ Public health center/health center branch/army doctor.

⑦ National Medical Center

⑧ Other (Please specify:)
